# *Zataria multiflora* Essential Oil–Silver Nanoparticle Hybrid Nanogel: A Dual‐Action Platform With Enhanced Antimicrobial Activity as a Promising Topical Antimicrobial System

**DOI:** 10.1155/cjid/2543116

**Published:** 2026-09-12

**Authors:** Mahmoud Osanloo, Ali Taghinezhad, Mohabbat Ansari, Fatemeh Noorbakhsh, Najmeh Namdar, Elham Zarenezhad

**Affiliations:** ^1^ Department of Medical Nanotechnology, School of Advanced Technologies in Medicine, Fasa University of Medical Sciences, Fasa, Iran, fums.ac.ir; ^2^ Noncommunicable Diseases Research Center, Fasa University of Medical Sciences, Fasa, Iran, fums.ac.ir; ^3^ Student Research Committee, Fasa University of Medical Sciences, Fasa, Iran, fums.ac.ir; ^4^ Department of Tissue Engineering, School of Advanced Technologies in Medicine, Fasa University of Medical Sciences, Fasa, Iran, fums.ac.ir

**Keywords:** antimicrobial platform, nanogel/hydrogel, silver nanoparticles (AgNPs), *Zataria multiflora*

## Abstract

The escalating global threat of antibiotic resistance underscores the urgent need for innovative antimicrobial strategies in wound management. In this regard, the integration of herbal therapeutics with noble metal nanoparticles, particularly silver, represents a promising combined approach for both infection control and tissue regeneration. In the present study, a novel nanogel system for advanced wound healing was developed through the incorporation of *Zataria multiflora* essential oil (ZMEO) with silver nanoparticles (AgNPs). Three carboxymethyl cellulose (CMC)–based hydrogel formulations were prepared, including an AgNP‐loaded hydrogel, a ZM‐loaded nanogel (ZM‐NGEL), and a hybrid nanogel (ZM‐Ag‐NGEL). Comprehensive physicochemical characterization demonstrated favorable formulation attributes. The primary nanoemulsions and AgNPs exhibited uniform nanoscale dimensions of 155 ± 7 and 53 ± 6 nm, respectively, along with strongly negative zeta potentials, indicative of excellent colloidal stability. Structural analyses using scanning electron microscopy (SEM) and Fourier transform infrared (FTIR) spectroscopy confirmed successful component integration and the formation of a polymeric network within the hybrid formulation. Rheological evaluation revealed pronounced shear‐thinning behavior and enhanced zero‐shear viscosity, most notably in the ZM‐Ag‐NGEL system. Functionally, the hybrid nanogel displayed superior, dose‐dependent, broad‐spectrum antibacterial activity against *Escherichia coli*, *Pseudomonas aeruginosa*, and *Staphylococcus aureus*, significantly outperforming the single‐component formulations. Collectively, these findings demonstrate that the ZM‐Ag‐NGEL system effectively combines the wound‐healing potential of *Zataria multiflora* with the potent antimicrobial activity of AgNPs within a stable hydrogel matrix, thereby positioning it as a promising antimicrobial platform for further investigation in topical and biomedical applications.

## 1. Introduction

Chronic wounds constitute a widespread and economically burdensome global healthcare challenge [1]. Each year, approximately 100 million individuals require wound care, with nearly 30 million affected by complex and persistent chronic skin wounds that demand advanced therapeutic interventions [1, 2]. Bacterial colonization and infection at the site of skin injury substantially delay the healing process and pose serious risks to patient health [3]. Consequently, the development of effective wound management strategies that simultaneously control microbial infection and maintain optimal wound moisture is of paramount importance for accelerating tissue repair [4]. Currently, composite hydrogels incorporating antibiotic agents have attracted considerable attention owing to their dual capacity to preserve a moist wound environment while preventing bacterial growth [5]. However, the widespread and often indiscriminate use of antibiotics has significantly intensified the problem of antimicrobial resistance, leading to the emergence of multidrug‐resistant bacterial strains [6]. Furthermore, achieving precise and sustained control over antibiotic release from hydrogel matrices remains technically challenging, thereby limiting their clinical efficacy in chronic wound treatment. Accordingly, the development of alternative antimicrobial agents represents a critical priority in the advancement of wound dressing technologies [7]. In this context, metal‐based nanomaterials and their oxides have demonstrated substantial efficacy in inhibiting bacterial proliferation and survival [8].

Over recent decades, extensive biomedical research has focused on metallic nanoparticles derived from noble metals, particularly silver and gold, due to their distinctive chemical, biological, and physical properties [9]. Among these, silver nanoparticles (AgNPs) have attracted pronounced scientific interest as potent antimicrobial agents [10, 11]. Although comprehensive understanding of their in vivo toxicity and biological fate remains incomplete, AgNPs have been widely employed across multiple sectors, including medicine, cosmetics, food packaging, textile surface modification, and environmental applications, largely because of their robust antibacterial activity [12, 13]. Various synthesis strategies have been developed for AgNP production, encompassing physical [14], chemical [15, 16], physicochemical [17], and biological approaches [18]. Notably, chemical reduction of silver salts, typically using reducing agents such as sodium citrate or sodium borohydride, has emerged as a preferred method owing to its operational simplicity, cost‐effectiveness, and reproducibility [19, 20].

Hydrogels are three‐dimensional, cross‐linked networks of hydrophilic polymers that are highly regarded as biomaterials due to their soft, tissue‐like elasticity, excellent biocompatibility, and capacity to regulate the diffusion of oxygen, nutrients, and bioactive compounds [21, 22]. AgNP‐embedded hydrogels have demonstrated strong antibacterial efficacy, leading to the commercialization of numerous silver‐based medical products, including wound dressings and vascular and urinary catheters [23, 24]. Antibacterial hydrogels may be applied directly to open wounds, either serving as carriers for antimicrobial agents or being intrinsically engineered to exhibit antimicrobial activity, thereby obviating the need for additional therapeutic compounds [25].

Within the framework of traditional Iranian medicine, a wide range of medicinal plants have been employed to enhance wound healing, among which *Zataria multiflora* holds a prominent position. This aromatic plant, belonging to the Lamiaceae family, is indigenous to regions of Iran, Afghanistan, and Pakistan. It contains a diverse array of phenolic and nonphenolic compounds, including thymol and carvacrol, which are associated with multiple pharmacological activities such as anticancer [26], antispasmodic [27], and antidiabetic effects [28]. Moreover, *Z. multiflora* exhibits pronounced anti‐inflammatory, antimicrobial, analgesic, anti‐apoptotic, and antioxidant properties, collectively enhancing its therapeutic value in wound healing applications [29]. Essential oils (EOs) are generally unsuitable for direct application due to poor aqueous solubility, rapid oxidation, and limited physicochemical stability. Consequently, they are commonly formulated as emulsions or nanoemulsions. Nano‐based delivery systems effectively address these limitations by improving stability and markedly enhancing skin permeability and bioavailability [30]. Notably, *Z. multiflora* has demonstrated enhanced antibacterial and wound‐healing efficacy when incorporated into nanotechnological formulations [31]. Therefore, the present study aims to evaluate and compare the antimicrobial performance of AgNP‐loaded hydrogels, *Z. multiflora* essential oil nanogels (ZM‐NGEL), their hybrid formulation (ZM‐Ag‐NGEL), and a blank hydrogel, with the goal of developing an advanced antibacterial nanogel system with potential for infection control applications.

## 2. Materials and Methods

### 2.1. Materials

Silver nitrate, trisodium citrate, Tween 20, and carboxymethyl cellulose (CMC) were purchased from Merck Chemicals (Germany). *Zataria multiflora* essential oil (ZMEO) was obtained from Zardband Pharmaceuticals Company (Iran).

### 2.2. Synthesis of AgNPs

A 2 mM silver nitrate (AgNO_3_) solution was prepared by dissolving 34 mg of AgNO_3_ in 100 mL of deionized water. Separately, a 20 mM trisodium citrate (C_6_H_5_Na_3_O_7_) solution was prepared. Subsequently, 5 mL of the silver nitrate solution was heated to boiling, after which 1 mL of the trisodium citrate solution was added dropwise under continuous stirring. The reaction mixture was stirred for 30 min, during which a visible color change was observed, indicating the formation of AgNPs.

The synthesized AgNPs were characterized using dynamic light scattering (DLS) to determine particle size (K‐One Nano Ltd., Korea) and zeta potential (Horiba SZ‐100, Japan).

The solution was then centrifuged, and the collected nanoparticle pellet was washed three times with deionized water to remove residual citrate and unreacted salts. After drying, the nanoparticles were weighed, and the estimated gravimetric synthesis yield was calculated using the following equation:

(1) Synthesis Efficiency %=Actual mass of nanoparticles collectedTheoretical mass of Ag×100%.

### 2.3. UV–Vis Spectroscopy of AgNPs

The optical properties of the synthesized AgNPs were evaluated using UV–visible spectroscopy over the wavelength range of 190–600 nm. The colloidal nanoparticle suspension was appropriately diluted with distilled water and scanned at room temperature. The maximum absorbance wavelength (*λ*_max) was recorded to confirm nanoparticle formation.

### 2.4. Preparation of ZM‐NGEL, Ag‐NGEL, and ZM‐Ag‐NGEL

ZMEO at a concentration of 1% w/v was mixed with varying concentrations of Tween 20 (0.5%, 1.0%, 1.5%, 2.0%, and 2.5% w/v) using a magnetic stirrer at 2000 rpm and room temperature. Distilled water was added dropwise to adjust the final volume to 5000 µL, and the mixture was stirred for an additional 40 min under identical conditions. The resulting nanoemulsions were analyzed by DLS (K‐One Nano Ltd.) to determine particle size distribution. The distribution width was evaluated using the SPAN value, calculated according to SPAN = (D90 − D10)/D50, where D10, D50, and D90 represent the particle diameters below which 10%, 50%, and 90% of the population are found, respectively. Nanoemulsions with particle sizes below 200 nm and SPAN values less than 1 were considered acceptable.

Only the formulation containing 1% w/v Tween 20 met these criteria; therefore, its zeta potential was measured, and it was selected as the optimized formulation for gel preparation.

### 2.5. Gel Formation

To prepare ZM‐NGEL, CMC was added to the optimized ZMEO nanoemulsion at a concentration of 3.5% w/v. The mixture was stirred overnight to ensure complete polymer hydration and chain swelling, resulting in uniform gel formation. A blank gel was prepared following the same procedure but without the addition of ZMEO.

### 2.6. Preparation of Nanocomposite Gels

Ag‐NGEL: This formulation was prepared by adding CMC (3.5% w/v) directly to an aqueous AgNP suspension with a concentration of 160 µg/mL, followed by overnight stirring.

ZM‐Ag‐NGEL: Dried citrate‐capped AgNPs were dispersed in the aqueous phase of the optimized ZMEO nanoemulsion to achieve a final concentration of 160 µg/mL prior to the addition of CMC (3.5% w/v). The resulting mixture was stirred overnight to obtain a homogeneous hybrid gel.

### 2.7. Field‐Emission Scanning Electron Microscopy (FE‐SEM)

The morphological features of AgNPs, ZM‐NGEL, and ZM‐Ag‐NGEL were examined using FE‐SEM. For AgNP imaging, a drop of the colloidal suspension was placed on an aluminum stub and air‐dried at ambient temperature, followed by sputter coating with a thin layer of gold. For nanogel samples, a suitable amount of each gel was allowed to dry at room temperature without freeze‐drying. The dried samples were mounted on scanning electron microscopy (SEM) stubs, gold‐coated, and subsequently imaged to evaluate surface morphology and nanoparticle distribution within the gel matrices.

### 2.8. Antibacterial Activity Test

The antibacterial activity of nanogels containing ZMEO (ZM‐NGEL), AgNPs (Ag‐NGEL), their combination (ZM‐Ag‐NGEL), and the blank gel was assessed using the AATCC 100 method with slight modifications. Defined amounts of each formulation (0.25, 0.5, and 1.0 g) were mixed with 4 mL of bacterial suspension (2 × 10^5^ CFU/mL) in test tubes, and the final volume was adjusted to 5 mL. The treated tubes were then incubated at 37°C for 1, 3, and 24 h under shaking conditions to ensure complete dissolution and uniform contact of the samples with the bacterial suspension. After each exposure period, 10 µL of each suspension was cultured on agar gel (Muller Hinton Agar plate) and incubated for 24 h. Subsequently, the number of colonies grown on the agar gel was counted and compared with the control group; the percentage of growth reduction was calculated using the following equation:

(2) CFU control=CFU sampleCFU control×100.

The concentration of ZMEO in ZM‐NGEL was 10,000 µg/mL, while the concentration of AgNPs in Ag‐NGEL was 160 µg/mL. The ZM‐Ag‐NGEL formulation contained both components at identical concentrations. Accordingly, the final concentrations of ZMEO in the test medium were 500, 1000, and 2000 µg/mL, while the corresponding AgNP concentrations were 8, 16, and 32 µg/mL. In the hybrid formulation, each dose level represented the simultaneous presence of both active agents at the specified concentrations. It should be noted that formal dose optimization studies (e.g., MIC determination) were not performed prior to these experiments; however, the selected dose range successfully demonstrated clear dose‐dependent activity and allowed effective comparison between formulations.

### 2.9. Statistical Analyses

All experiments were performed in triplicate, and the results are presented as mean ± standard deviation (SD). Normality was assessed using the Shapiro–Wilk test, which indicated a non‐normal distribution of antibacterial data. Consequently, nonparametric statistical analyses were applied. Differences among groups were evaluated using the Kruskal–Wallis test, followed by Dunn’s post hoc test with Bonferroni correction for multiple comparisons.

## 3. Results

### 3.1. Synthesis Efficiency of AgNPs

The synthesis efficiency of AgNPs was determined by centrifugation‐based isolation of the nanoparticles, followed by oven drying and gravimetric analysis. The experimentally obtained nanoparticle mass was compared with the theoretical silver content derived from the AgNO_3_ precursor. Based on gravimetric analysis of the washed nanoparticle pellet, the estimated synthesis yield was calculated to be approximately 93%. This value should be considered an approximate recovery yield rather than a direct measure of silver conversion efficiency.

Considering the 5:1 volumetric ratio of the precursor solutions, the effective concentration of silver nitrate in the final mixture was 0.283 mg/mL. This value was multiplied by 0.635, corresponding to the silver mass fraction in AgNO_3_, yielding a pure silver concentration of 0.180 mg/mL. After accounting for the 93% synthesis efficiency, the final concentration of AgNPs in the 6‐mL solution was calculated to be 167 µg/mL.

### 3.2. Size and Zeta Potential of ZM‐NGEL Primary Nanoemulsion and AgNPs

**FIGURE 1:**
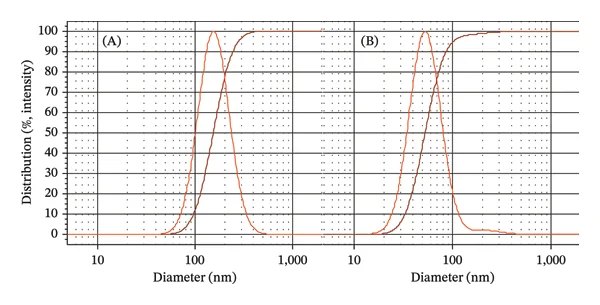
DLS profiles of A: ZM‐NGEL primary nanoemulsion and B: AgNPs.

The particle size distributions of the synthesized nanostructures, as determined by DLS analysis, are presented in Figure 1. The ZM‐NGEL primary nanoemulsion exhibited an average particle size of 155 ± 7 nm with a SPAN value of 0.94, reflecting a highly homogeneous nanoemulsion system. In contrast, AgNPs displayed a smaller average particle size of 53 ± 6 nm and a SPAN value of 0.98, confirming the successful synthesis of monodisperse nanoparticles with a narrow size distribution. The consistently low SPAN values observed for both systems indicate the formation of uniform colloidal dispersions suitable for pharmaceutical applications.

**FIGURE 2:**
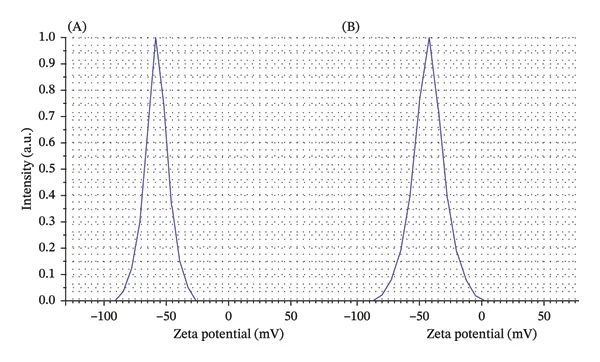
Zeta potential profiles of A: ZM‐NGEL primary nanoemulsion and B: AgNPs.

The surface charge characteristics and colloidal stability of the nanostructures were evaluated through zeta potential measurements, as illustrated in Figure 2. The ZM‐NGEL primary nanoemulsion exhibited a zeta potential of −58 ± 2 mV, while AgNPs showed a zeta potential of −42 ± 2 mV. These strongly negative values indicate excellent electrostatic stability and a low tendency toward aggregation. The high negative surface charge of the ZM‐NGEL nanoemulsion is attributed primarily to ionizable phenolic and other oxygen‐containing constituents present in ZMEO. In contrast, the negative zeta potential of AgNPs is consistent with citrate‐mediated synthesis, where adsorbed citrate ions act as surface‐stabilizing agents.

**FIGURE 3:**
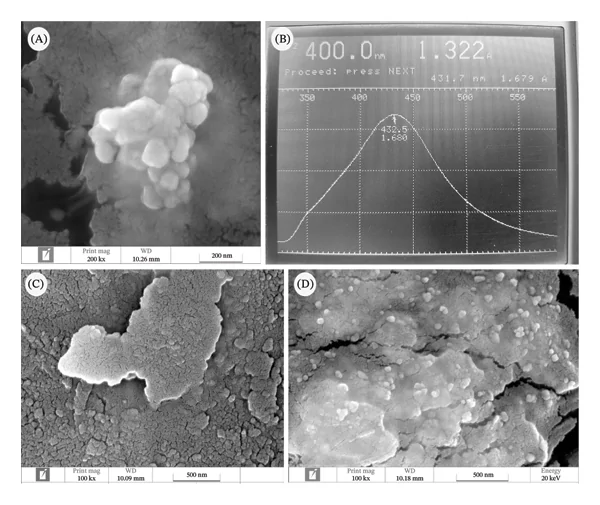
(A) FE‐SEM image of AgNPs; (B) UV–Vis spectrum of AgNPs; (C) SEM image of ZM‐NGEL; (D) SEM image of ZM‐Ag‐NGEL.

The morphological characteristics of the synthesized AgNPs and the prepared nanogels were investigated using SEM and UV–visible spectroscopy, as illustrated in Figure 3. As shown in Figure 3A, the AgNPs exhibited a predominantly spherical morphology, with a slight tendency toward the formation of small aggregates, a phenomenon commonly observed in metallic nanoparticles due to their high surface energy at the nanoscale. The UV–visible spectrum of the AgNPs (Figure 3B) displayed a distinct surface plasmon resonance (SPR) peak at approximately 432 nm, thereby confirming the successful formation of AgNPs and indicating their stable colloidal nature.

The SEM micrograph of the ZM‐NGEL formulation (Figure 3C) revealed a granular, particle‐like surface morphology, which can be attributed to the presence of ZMEO‐loaded droplets embedded within the nanogel matrix. Following the incorporation of AgNPs into the nanogel structure, the SEM image of the ZM‐Ag‐NGEL formulation (Figure 3D) showed bright, spherical AgNPs uniformly distributed across the gel surface, confirming the successful loading and homogeneous dispersion of the nanoparticles within the nanogel network. It should be noted that SEM images were obtained from air‐dried samples and therefore may not fully represent the native hydrated microstructure of the nanogels.

**FIGURE 4:**
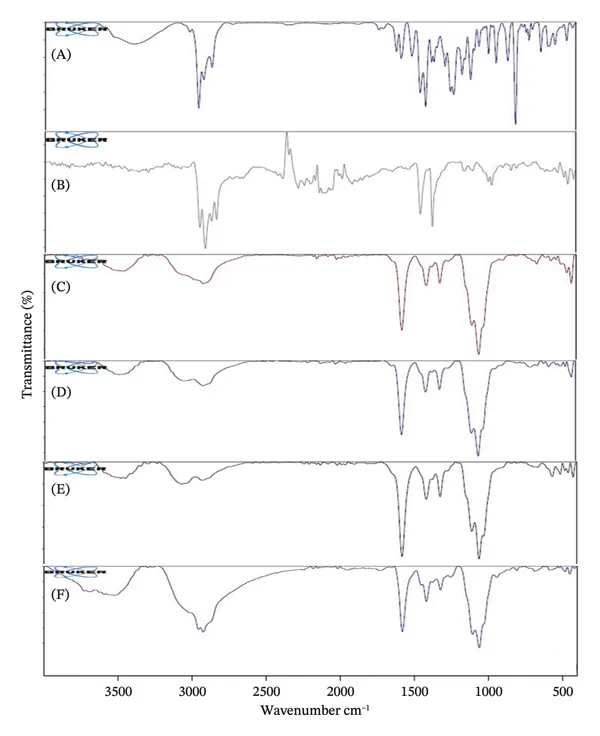
ATR‐FTIR of (A) ZMEO, (B) AgNPs, (C) blank gel, (D) ZM‐NGEL, (E) Ag‐NGEL, and (F) ZM‐Ag‐NGEL.

The Fourier transform infrared (FTIR) spectrum of ZMEO (Figure 4A) exhibited a broad absorption band in the range of 3300–3650 cm^−1^, which is attributed to O–H stretching vibrations of phenolic compounds resulting from hydrogen bonding. The characteristic band observed at 3019 cm^−1^ corresponds to C=C–H stretching vibrations, while the bands at 2959, 2925, and 2869 cm^−1^ are associated with symmetric and asymmetric C–H stretching vibrations of methyl (CH_3_) groups. A strong absorption band at 1738 cm^−1^ confirms the presence of carbonyl functional groups, which may arise from ester bonds and carboxylic acid moieties, with additional contributions observed around 1706 cm^−1^. The band at 1457 cm^−1^ is attributed to C═C–C ring vibrations of aromatic structures. Furthermore, the strong absorption at 1018 cm^−1^ corresponds to C–O stretching vibrations, while the peak at 810 cm^−1^ is associated with C–H bending vibrations of the aromatic ring, consistent with previous reports [32, 33].

As shown in Figure 4B, the FTIR spectrum of the synthesized AgNPs displayed a broad band between 3200 and 3600 cm^−1^, which is assigned to O–H stretching vibrations arising from hydrogen bonding. The absorption bands at 2950, 2914, and 2870 cm^−1^ correspond to C–H stretching vibrations associated with sp^3^‐hybridized alkane structures. The peak observed at 1455 cm^−1^ is attributed to the asymmetric stretching of carboxylate groups (–COO^−^), which is consistent with the citrate ions present on the nanoparticle surface as a capping agent. Accordingly, the band at 1374 cm^−1^ corresponds to symmetric carboxylate stretching, further confirming the presence of citrate species coordinated to the AgNPs [34]. Since metal nanoparticles generated in solution require stabilization to prevent aggregation caused by van der Waals attractive forces, the presence of hydroxyl and carboxylate groups, as evidenced by FTIR analysis, confirms the role of citrate ions as a capping and stabilizing agent.

The FTIR spectrum of the blank gel (Figure 4C) exhibited a broad absorption band between 3297 and 3477 cm^−1^, which is attributed to hydroxyl groups engaged in hydrogen bonding among Tween, CMC, and water molecules. The absorption band at 3297 cm^−1^ is associated with C=C–H stretching vibrations, while the band at 2927 cm^−1^ corresponds to C–H stretching vibrations. A strong absorption band at 1582 cm^−1^ is attributed to the asymmetric stretching vibration of the COO^−^ functional group of CMC, confirming the presence of this moiety in the gel matrix. The intense peaks observed at 1108 and 1062 cm^−1^ are assigned to C–O stretching vibrations [35].

The FTIR spectrum of the *Zataria multiflora* gel (Figure 4D) exhibited a characteristic broad peak at 3498 cm^−1^, which is attributed to hydroxyl groups involved in hydrogen bonding among Tween, EO components, CMC, and water. The band at 3059 cm^−1^ can be assigned to =C=C–H stretching vibrations, while the absorption at 2929 cm^−1^ corresponds to C–H stretching vibrations arising from EO, Tween, and CMC. A pronounced absorption band at 1582 cm^−1^ is associated with the COO^−^ group of CMC [36]. The sharp and intense peaks at 1108 and 1062 cm^−1^ correspond to C–O stretching vibrations.

As illustrated in Figure 4E, the FTIR spectrum of the AgNPs gel exhibited a strong and broad absorption band at 3449 cm^−1^, which is attributed to hydroxyl groups engaged in hydrogen bonding among Tween, CMC, citrate ions, and AgNPs. The band at 3066 cm^−1^ corresponds to C=C–H stretching vibrations, while the absorption at 2931 cm^−1^ is associated with C–H stretching vibrations in Tween, CMC, and citrate. The peak at 1581 cm^−1^ is related to glucose ring vibrations, a structural feature characteristic of CMC. The absorption band at 1324 cm^−1^ is attributed to C–O–H bending vibrations or –CH_2_ wagging modes of the polysaccharide backbone, confirming the presence of CMC within the gel matrix. [37]. The band at 1111 cm^−1^ corresponds to C–O stretching vibrations. Collectively, these spectral features confirm the successful incorporation and stability of AgNPs within the gel network [38].

The FTIR spectrum of the AgNPs gel containing ZMEO (Figure 4F) exhibited a very broad absorption band spanning 3299–3694 cm^−1^, which is attributed to hydroxyl groups involved in hydrogen bonding between alcohols and phenolic compounds present in the EO and the gel matrix. C–H stretching vibrations were observed at approximately 2957 and 2925 cm^−1^. The absorption band around 1738 cm^−1^ corresponds to carbonyl functional groups originating from organic constituents of the EO [39]. The C–O stretching vibrations observed between 1063 and 1108 cm^−1^ are attributed to polysaccharides or other oxygen‐containing functional groups present in the gel matrix or the EO. Collectively, FTIR analysis corroborates the successful formulation of a physically crosslinked hybrid nanocomposite gel with preserved chemical integrity of its components.

### 3.3. Rheological Properties

**FIGURE 5:**
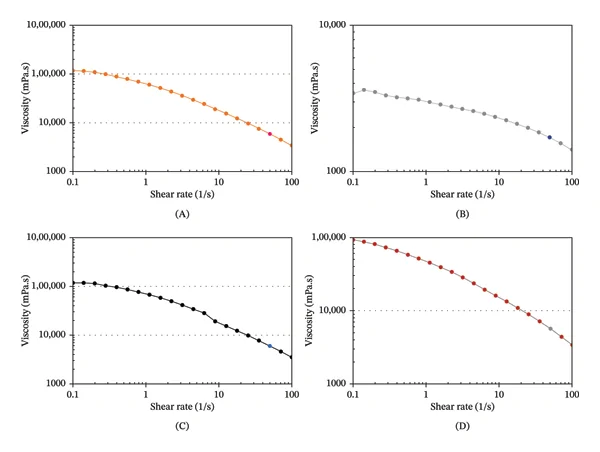
Rheological properties of the prepared nanogels: (A) blank gel, (B) ZM‐NGEL, (C) Ag‐NGEL, and (D) ZM‐Ag‐NGEL.

The viscosity–shear rate profiles (Figure 5A–D) indicate that all gel formulations exhibit pronounced shear‐thinning behavior, characterized by a systematic decrease in viscosity with increasing shear rate. The incorporation of ZM‐NGEL, AgNPs (Ag‐NGEL), or their combination (ZM‐Ag‐NGEL) markedly increased the zero‐shear viscosity relative to the blank gel, indicating that these active constituents reinforce the polymeric network and enhance its structural integrity under static or low‐shear conditions. Notably, the hybrid ZM‐Ag‐NGEL formulation displayed the greatest viscosity enhancement at low shear rates, suggesting an enhanced interaction between *Z. multiflora* constituents and AgNPs that further strengthens the gel matrix. Collectively, these findings demonstrate that the inclusion of bioactive components, particularly in combination, effectively modulates the rheological behavior of the system by increasing resistance to deformation at low shear while preserving the characteristic shear‐responsive flow essential for practical application.

### 3.4. Antimicrobial Efficacy

**FIGURE 6:**
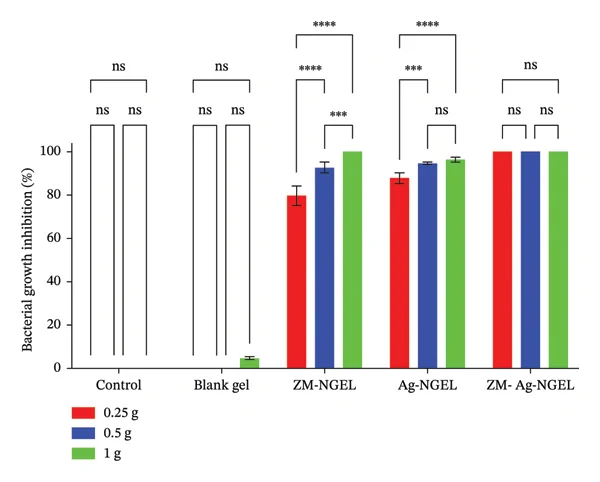
Antibacterial activity of ZM‐NGEL, Ag‐NGEL, and ZM‐Ag‐NGEL against *E. coli* at three dose levels (0.25, 0.5, and 1.0 g), corresponding to final concentrations of 500, 1000, and 2000 µg/mL ZMEO and 8, 16, and 32 µg/mL AgNPs. Control: untreated bacterial suspension (without any formulation). Blank gel: CMC hydrogel without ZMEO or AgNPs.

**FIGURE 7:**
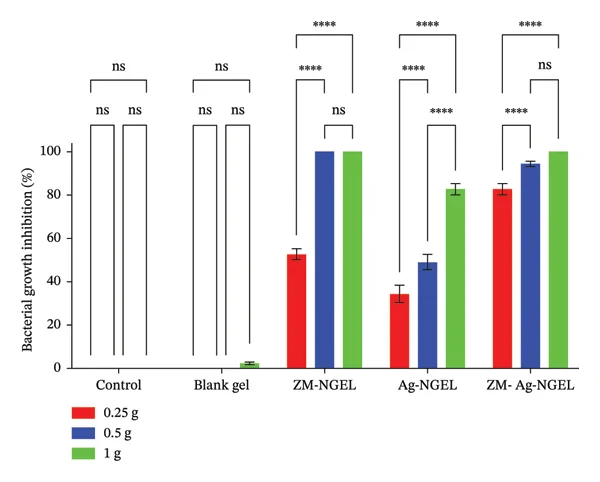
Antibacterial activity of ZM‐NGEL, Ag‐NGEL, and ZM‐Ag‐NGEL against *P. aeruginosa* at three dose levels (0.25, 0.5, and 1.0 g), corresponding to final concentrations of 500, 1000, and 2000 µg/mL ZMEO and 8, 16, and 32 µg/mL AgNPs. Control: untreated bacterial suspension (without any formulation). Blank gel: CMC hydrogel without ZMEO or AgNPs.

**FIGURE 8:**
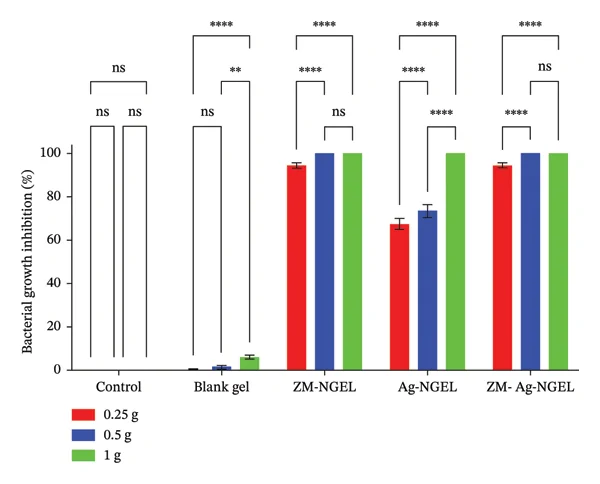
Antibacterial activity of ZM‐NGEL, Ag‐NGEL, and ZM‐Ag‐NGEL against *S. aureus* at three dose levels (0.25, 0.5, and 1.0 g), corresponding to final concentrations of 500, 1000, and 2000 µg/mL ZMEO and 8, 16, and 32 µg/mL AgNPs. Control: untreated bacterial suspension (without any formulation). Blank gel: CMC hydrogel without ZMEO or AgNPs.

The antimicrobial activity of the synthesized hydrogel formulations was assessed against three clinically relevant bacterial pathogens, namely *Escherichia coli*, *Pseudomonas aeruginosa*, and *Staphylococcus aureus* (Figures 6, 7, and 8). Across all tested strains, the results revealed a clear and consistent dose‐dependent antibacterial response. As anticipated, the control and blank gel formulations, which lacked active antimicrobial agents, exhibited no detectable inhibitory effect against any of the evaluated microorganisms.

Among the active formulations, the hybrid hydrogel incorporating both ZMEO and AgNPs (ZM‐Ag‐NGEL) demonstrated the highest antibacterial efficacy against all three pathogens, consistently surpassing the inhibitory performance of the single‐component formulations, namely, ZM‐NGEL and Ag‐NGEL. This superior activity indicates a pronounced enhanced interaction between *Z. multiflora* bioactive compounds and AgNPs, resulting in broad‐spectrum antibacterial activity against both Gram‐negative bacteria (*E. coli* and *P. aeruginosa*) and the Gram‐positive bacterium *S. aureus*.

Furthermore, a positive concentration–response relationship was observed across all treatment groups, with increasing hydrogel mass from 0.25 to 1.0 g leading to progressively enhanced antibacterial effects. Notably, the ZM‐Ag‐NGEL formulation at the highest concentration exhibited particularly strong inhibition, exceeding the expected additive effects of its individual components and underscoring the presence of enhanced antimicrobial mechanisms. Importantly, even at lower concentrations, ZM‐Ag‐NGEL retained substantial antibacterial activity, highlighting its potential effectiveness at reduced dosages. Such efficiency may translate into lower toxicity risks and improved cost‐effectiveness in practical applications.

Taken together, these results establish ZM‐Ag‐NGEL as a highly promising broad‐spectrum antimicrobial platform with potential applications in wound dressings, antibacterial surface coatings, and infection control strategies.

## 4. Discussion

Bacterial infections caused by *E. coli*, *P. aeruginosa*, and *S. aureus* constitute major public health concerns owing to their high prevalence, remarkable adaptability, and rapidly increasing resistance to conventional antibiotics [40]. *E. coli*, frequently associated with urinary tract infections, gastrointestinal diseases, and foodborne outbreaks, has demonstrated a pronounced capacity to evolve into highly virulent and multidrug‐resistant strains, such as enterohemorrhagic *E. coli* (EHEC) [41]. Similarly, *P. aeruginosa* is a notorious opportunistic pathogen responsible for numerous hospital‐acquired infections, posing a particularly serious threat to immunocompromised individuals because of its intrinsic resistance mechanisms and ability to form persistent biofilms [42]. *S. aureus*, including methicillin‐resistant *S. aureus* (MRSA), is implicated in a wide spectrum of infections, ranging from mild skin conditions to severe, life‐threatening diseases such as sepsis and pneumonia. Collectively, these pathogens significantly contribute to the global burden of antimicrobial resistance, underscoring the urgent need for innovative therapeutic strategies and alternative antimicrobial agents [43, 44].

The antimicrobial activity of EOs against a broad range of microorganisms has been well documented. Their potent antibacterial effects are largely attributed to phenolic constituents, which interact with phospholipid components of bacterial cell membranes, thereby increasing membrane permeability, inducing leakage of essential intracellular contents, and disrupting key enzymatic systems [44, 45]. Despite their promising bioactivity, the clinical applicability of EOs is often limited by their volatility, poor aqueous solubility, and reduced stability [46]. Consequently, transforming EOs into nanostructured delivery systems, including nanoemulsions, polymeric nanoparticles, and silver‐based nanocarriers, has emerged as an effective approach to overcome these limitations and enhance antimicrobial efficacy [47]. In this context, Osanloo et al. evaluated the antibacterial activity of ZMEO and reported IC_50_ values of approximately 129, 155, 717, and 140 μg/mL against *S. aureus*, *E. coli*, *P. aeruginosa*, and *K. pneumoniae*, respectively [48]. Subsequent formulation of ZMEO as a nanoemulsion with an average droplet size of 155 ± 7 nm resulted in a substantial enhancement of its antibacterial performance [49]. Moreover, ZM‐based hydrogels have been shown to significantly promote wound healing in animal models. In particular, ZM‐GEL at a concentration of 4% markedly improved wound contraction and tissue regeneration in surgical wounds compared with lower concentrations and control groups [50].

Hydrogels are three‐dimensional, hydrophilic polymer networks capable of absorbing and retaining large amounts of water, thereby forming a moist, semisolid matrix highly suitable for wound healing applications. By maintaining optimal hydration at the wound site, hydrogels facilitate tissue regeneration and can be further functionalized with antimicrobial or bioactive agents to accelerate healing and prevent infection [51, 52]. In previous studies, hydrogels incorporating AgNPs, synthesized through the reduction of silver nitrate, demonstrated strong antibacterial activity against *E. coli* and *S. aureus*, with higher silver concentrations producing greater bacterial inhibition [53]. Polysaccharide‐based hydrogels containing AgNPs have likewise been confirmed to possess considerable potential for the treatment of infected wounds [54]. Qi et al. developed an antibacterial wound dressing by incorporating AgNPs into a polydopamine‐based hydrogel matrix, demonstrating enhanced antibacterial activity and wound healing efficacy [55]. Additionally, AgNP‐loaded hydrogels have exhibited excellent swelling capacity and water‐retention properties while preserving the inherent hydrophilicity of CMC. The incorporation of green‐synthesized AgNPs has further enabled sustained nanoparticle release for up to 8 h, thereby ensuring prolonged antimicrobial activity and enhanced wound‐healing efficacy [56].

AgNP‐loaded hydrogels are known to exert potent antimicrobial effects against a broad spectrum of microorganisms, including both Gram‐positive and Gram‐negative bacteria, as well as drug‐resistant strains such as *S. aureus* and *P. aeruginosa*. Their antibacterial activity primarily arises from two complementary mechanisms: first, the controlled release of silver ions (Ag^+^), which disrupt bacterial membranes and interfere with essential enzymatic processes; and second, the direct interaction of AgNPs with bacterial cell surfaces, leading to structural damage and cell death. In addition to their antibacterial properties, such hydrogels also display antifungal activity and are particularly well suited for applications requiring both antimicrobial efficacy and biocompatibility, including wound dressings and implant coatings [57].

Hydrogels formulated with ZMEO rely on the bioactivity of naturally occurring phytochemicals, particularly thymol and carvacrol, to exert antimicrobial effects. These compounds compromise microbial membrane integrity and disrupt cellular respiration pathways [58]. When incorporated into nanostructured systems such as nanogels or nanoemulsions, the antimicrobial efficacy of *Z. multiflora* is markedly enhanced, with reported inhibition levels reaching up to 79% against fungal species and substantial antibacterial activity against *E. coli* and *S. aureus*. Beyond their antimicrobial action, these nanoformulations also exhibit pronounced antioxidant and anti‐inflammatory properties, which collectively support tissue regeneration and accelerate wound repair [59, 60].

Notably, the combined formulation of *Z. multiflora* and AgNPs (ZM‐Ag‐NGEL) achieved complete bacterial growth inhibition at the highest tested concentration of 1.0 g. This pronounced antibacterial effect reflects an enhanced interaction between AgNPs and the bioactive constituents of ZMEO, which further strengthens the gel matrix. Silver ions increase bacterial membrane permeability, thereby facilitating deeper penetration of thymol and carvacrol into bacterial cells. Such combined interactions between metal nanoparticles and plant‐derived bioactives have been widely reported to enhance antimicrobial efficacy and contribute to overcoming bacterial resistance mechanisms [61–63].

Overall, the present findings demonstrate that the incorporation of ZMEO and AgNPs into a unified nanogel platform markedly enhances antibacterial performance. This hybrid nanocomposite shows strong promise for biomedical applications, particularly in topical antimicrobial formulations, where broad‐spectrum antimicrobial activity is a critical requirement.

### 4.1. Study Limitations and Future Directions

A key limitation of this study is its exclusive focus on physicochemical characterization, rheological properties, and in vitro antibacterial activity. We did not conduct direct cytotoxicity assays (e.g., MTT), cell viability assessments, or biocompatibility evaluations, nor did we perform cell migration or in vivo wound‐healing experiments. Consequently, while the ZM‐Ag‐NGEL formulation demonstrates potent antibacterial effects and favorable structural attributes, its safety, cytocompatibility, and therapeutic efficacy remain unconfirmed. Furthermore, we did not perform formal synergy analyses, such as checkerboard assays with FIC index calculations, to determine whether the enhanced antibacterial action reflects true synergy, additivity, or independent effects.

To address these gaps, future work will comprehensively evaluate cytocompatibility using relevant cell lines (e.g., fibroblasts or keratinocytes) and assess in vivo wound‐healing performance and systemic toxicity in appropriate animal models prior to clinical translation. Concurrently, systematic synergy studies employing checkerboard titrations and FIC indices (e.g., via CompuSyn software) are planned to precisely define the ZMEO–AgNP interaction and guide formulation optimization for maximal efficacy.

## 5. Conclusions

In this study, a novel hybrid hydrogel (ZM‐Ag‐NGEL) was successfully developed through the enhanced integration of ZMEO and AgNPs. The resulting formulation exhibited optimal nanoscale characteristics, excellent colloidal stability, and clear evidence of molecular interactions within the gel network. Rheological analysis revealed enhanced structural integrity, which correlated with significantly improved, dose‐dependent antimicrobial activity against both Gram‐positive and Gram‐negative bacteria compared with the individual component formulations. These results establish ZM‐Ag‐NGEL as a highly promising antibacterial platform that warrants further biological evaluation, including comprehensive biocompatibility and cytotoxicity assessments, to explore its potential for infection control and tissue repair applications.

## Author Contributions

The conception, design, of the study was done by Mahmoud Osanloo and Elham Zarenezhad. The first draft of the manuscript was written by Mahmoud Osanloo, Ali Taghinezhad, Mohabbat Ansari, Fatemeh Noorbakhsh, Najmeh Namdar, and Elham Zarenezhad.

## Funding

This study was supported by Noncommunicable Diseases Research Center, Fasa University of Medical Sciences (Grant No. 403185).

## Disclosure

All authors approved the final manuscript.

## Conflicts of Interest

The authors declare no conflicts of interest.

## Data Availability

The data that support the findings of this study are available from the corresponding author upon reasonable request.
